# TRENDS AND VARIATION OF MEDICARE ADVANTAGE AMONG LONG-STAY NURSING HOME RESIDENTS

**DOI:** 10.1093/geroni/igae098.0224

**Published:** 2024-12-31

**Authors:** Hyunkyung Yun, Cyrus Kosar

**Affiliations:** Brown University, Providence, Rhode Island, United States; Brown University, Providence, Rhode Island, United States

## Abstract

Despite the large growth of the Medicare Advantage (MA) to now comprise over 50% of all Medicare beneficiaries, the role of MA in long-term care has been understudied. We examined trends in MA enrollment among long-stay nursing home (NH) residents and geographic variation in these trends (2010 to 2021). We compared the individual and NH characteristics between beneficiaries of traditional Medicare (TM), general MA, and MA plans specialized for long-term care, Institutional Special Needs Plans (I-SNPs). Among long-stay residents, MA enrollment grew from 13.0% in 2010 to 31.6% in 2021, while MA enrollment increased from 23.6% to 40.8% for all other Medicare beneficiaries. I-SNP enrollment grew substantially; in 2010, 34 I-SNPs enrolled 15.7% of MA long-stay residents. By 2021, 126 I-SNPs enrolled 30.8% of them. The individual- and facility- characteristics for TM and non-I-SNP MA enrollees were broadly comparable. I-SNP enrollees, however, were more likely to be Black (20.4% of residents vs 14.5% for TM, 13.5% for other MA) and dually enrolled in Medicaid (I-SNP 97.7%, TM 83.6%, other MA 83.1%). I-SNP enrollees were more likely to be in NH with 1-2 (low) star-ratings (I-SNP 38.7%, TM 38.4%, other MA 38.2%) and less likely to reside in facilities with 4-5 (high) star-ratings (I-SNP 38.7%, TM 42.1%, other MA 42.4%). MA enrollment has grown substantially among long-stay residents. As new models such as I-SNPs become more prevalent, future research and monitoring are needed to ensure that the care needs of this high-need population are being adequately met.

